# The role of circadian clock-controlled mitochondrial dynamics in diabetic cardiomyopathy

**DOI:** 10.3389/fimmu.2023.1142512

**Published:** 2023-05-05

**Authors:** Zhenshuai Jin, Yanwei Ji, Wating Su, Lu Zhou, Xiaojing Wu, Lei Gao, Junfan Guo, Yutong Liu, Yuefu Zhang, Xinyu Wen, Zhong-Yuan Xia, Zhengyuan Xia, Shaoqing Lei

**Affiliations:** ^1^ Department of Anesthesiology, Renmin Hospital of Wuhan University, Wuhan, China; ^2^ Department of Anesthesiology, Affiliated Hospital of Guangdong Medical University, Zhanjiang, China; ^3^ Faculty of Chinese Medicine, State Key Laboratory of Quality Research in Chinese Medicine, Macau University of Science and Technology, Taipa, Macao SAR, China

**Keywords:** diabetic cardiomyopathy, clock circadian, mitochondrial dynamics, mitochondrial fusion, mitochondrial fission

## Abstract

Diabetes mellitus is a metabolic disease with a high prevalence worldwide, and cardiovascular complications are the leading cause of mortality in patients with diabetes. Diabetic cardiomyopathy (DCM), which is prone to heart failure with preserved ejection fraction, is defined as a cardiac dysfunction without conventional cardiac risk factors such as coronary heart disease and hypertension. Mitochondria are the centers of energy metabolism that are very important for maintaining the function of the heart. They are highly dynamic in response to environmental changes through mitochondrial dynamics. The disruption of mitochondrial dynamics is closely related to the occurrence and development of DCM. Mitochondrial dynamics are controlled by circadian clock and show oscillation rhythm. This rhythm enables mitochondria to respond to changing energy demands in different environments, but it is disordered in diabetes. In this review, we summarize the significant role of circadian clock-controlled mitochondrial dynamics in the etiology of DCM and hope to play a certain enlightening role in the treatment of DCM.

## 1 Introduction

The incidence of diabetes mellitus is increasing, and now more than 350 million people are reported to suffer from diabetes worldwide (1). The population and condition of diabetes mellitus have become more and more juvenile and complicated (2). Patients with diabetes may develop cardiovascular complications, especially diabetic cardiomyopathy (DCM), which is prone to heart failure with preserved ejection fraction (HFpEF). This type of heart failure was first reported in 1972 in patients with type 2 diabetes, who had no risk factors for heart failure, such as hypertension and coronary artery disease (3). Subsequent clinical and experimental studies gradually revealed the main pathophysiological mechanisms of DCM, such as inflammation, lipid accumulation, myocardial fibrosis, cardiac hypertrophy, cardiac apoptosis, microvascular damage, etc. (4, 5) The main clinical features of DCM are cardiac hypertrophy, diastolic dysfunction, and myocardium stiffing (6). At the late stage of DCM, the myocardial systolic function is also affected, leading to dilated cardiomyopathy (6). However, the pathophysiological mechanisms of DCM remain complex and unclear.

Increasing studies suggest the involvement of dysfunctional mitochondria in the pathophysiology of DCM. The dysfunctional mitochondria result in myocardial metabolic disorders, oxidative stress, Ca^2+^ overload, myocardial systolic/diastolic dysfunction, and myocardial stiffness (7, 8). Mitochondria are the centers of energy metabolism that are extremely essential for maintaining the function of the heart, an organ with high energy requirements. Mitochondria are highly dynamic in response to environmental changes through mitochondrial dynamics, including mitochondrial fusion and fission. Noteworthily, mitochondrial dynamics has a circadian rhythm throughout the day. The circadian rhythm of mitochondrial dynamics is regulated by circadian clock genes that mediate the expression of mitochondrial dynamic molecules and affect mitochondrial morphology and function (9). When circadian clock genes are mutated or disrupted, mitochondrial dynamics may lose circadian rhythm and become disordered, resulting in insulin resistance, cardiac lipotoxicity, excessive production of mitochondrial reactive oxidative species (ROS), mitochondrial Ca^2+^ mishandling, decreased mitochondrial membrane potential (MMP), impaired mitophagy, and endoplasmic reticulum (ER) stress, which are associated with the pathophysiology of DCM (9–11). Thus, recent advances in understanding clock-controlled mitochondrial dynamics and its implication for the pathophysiology of DCM may open up novel therapeutic avenues.

## 2 Mitochondrial dynamics

Mitochondria are highly dynamic organelles, constantly changing their morphology, from tubular (fusion) to fragmented (fission). The balance between mitochondrial fusion and division is important for the proper functioning of cells. Disruptions in mitochondrial dynamics affect mitochondrial morphology and function, leading in the development of disease, DCM.

### 2.1 Mitochondrial fusion

Mitochondria have an outer mitochondrial membrane (OMM) and an inner mitochondrial membrane (IMM) (12). The progress of mitochondrial fusion includes OMM fusion and IMM fusion. Mitofusin1/2 (MFN1/2), belongs to the family of GTPases, and primarily orchestrates OMM fusion (12). As a transmembrane protein anchored to the OMM, MFN1/2 contains the N-terminal GTPase domain and heptad-repeat regions (HR1 and HR2) (13). When the tips of two mitochondria meet in the cytoplasm, MFN1/2 as a tether interacts with another mitochondrion, and forms the MFN homodimer or heterodimer, then alters the conformation of the HR2 region depending on GTPase, resulting in the fusion of OMM. In fact, MFN1 plays a leading role in the process of mitochondrial fusion, the role of MFN2 remains elusive, which primarily participated in the site of OMM interacting with other organelles (particularly the ER) (14, 15).

**Figure 1:**
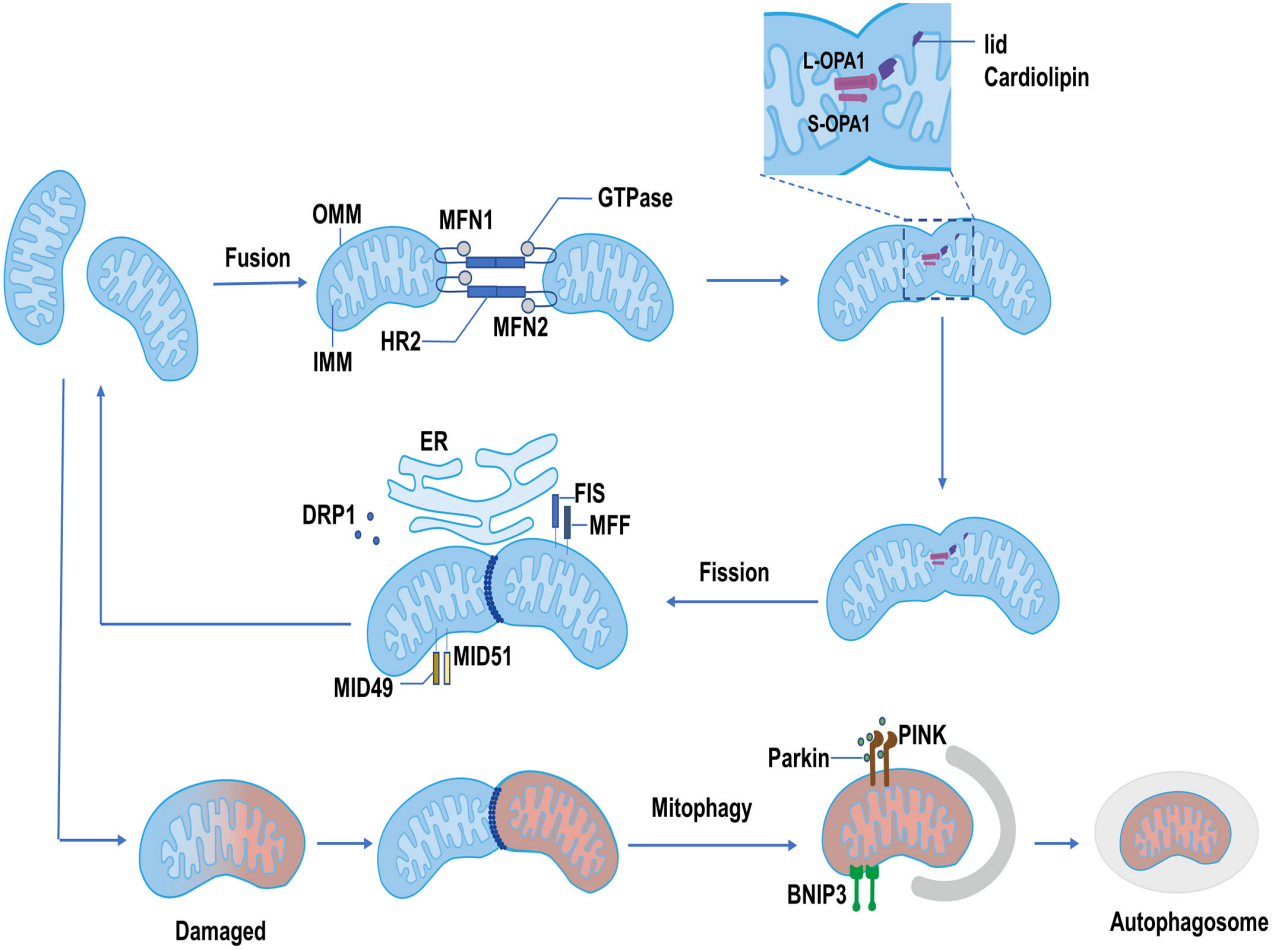
The process of mitochondrial dynamics. Mitochondrial fusion: MFN1 and MFN2 form homodimer or heterodimer, then alter the conformation of the HR2 region depending on GTPase, resulting in the fusion of OMM. IMM fusion is mainly orchestrated by OPA1. The L-OPA1 interacts with lid cardiolipin and facilitates the fusion of the IMM. The S-OPA1 interacts with L-OPA1 and promotes IMM fusion. Mitochondrial fission: The dephosphorylated DRP1 is recruited at the mitochondrial membrane by its receptors, mainly including FIS and MFF. MID49 and MID51 also recruit DRP1 when the MFF/FIS1 is not available. Recruited DRP1 combined with the receptor, forming a ring-like structure to shear the mitochondria and promote the completion of mitochondrial fission. Mitophagy: Damaged mitochondria are degraded by autophagosomes through PINK1/Parkin and BNIP3 pathways.

After OMM fusion, the IMM subsequently starts to fusion, and optic atrophy (OPA1) as a pivotal factor mainly participated in the process of IMM fusion. OPA1 consists of long OPA1 (L-OPA1) and short OPA1 (S-OPA1). L-OPA1 interacts with cardiolipin on the IMM to facilitate the fusion of the IMM. S-OPA1, which is produced by the degradation of L-OPA1 by proteolytic enzymes OMA1 and YME1-like ATPase (YME1L) (12), is mainly to promote mitochondrial fusion by assisting L-OPA1. However, when S-OPA1 over-accumulates, it will suppress the role of L-OPA1 (12), leading to mitochondrial division and disruption of mitochondrial dynamics (**Figure 1**) (16). OPA1 is not only participated in IMM fusion but also plays an important role in the remodeling of mitochondrial cristae, which is the site of oxidative phosphorylation (OXPHOS) and ATP synthase (17). The left-turned assemblies at the cristate (the structure of OPA1 is involved in the right- or left-turned helical assemblies) could prevent cytochrome C entering from the matrix into the intermembranous mitochondria by tightening mitochondrial crista and diminishing crista lumen. When OPA1 is reduced or destroyed, a large amount of cytochrome C enters into the intermembrane, then enters into the cytoplasm through the permeable out membrane, and finally induces cell apoptosis (12, 18). Overall, OPA1 has extensive effects on mitochondrial function, and different aspects of its function need to be further refined.

### 2.2 Mitochondrial fission

The proteins involved in the mitochondrial fission process mainly include dynamin-related protein1 (DRP1) and mitochondrial fission factor (MFF)/fission protein 1 (FIS1). DRP1 performs a critical role in mitochondrial fission by translocation to mitochondrial membranes and binding to receptors. In the cytoplasm, the activity of DRP1 is regulated by many factors, such as cAMP-dependent serine/threonine-specific protein kinase A (PKA). PKA phosphorylates the tryptophan of DRP1, stabilizing DRP1 in the cytoplasm and promoting mitochondrial elongation (19, 20). Besides, DRP1 is dephosphorylated by Ca^2+^-dependent phosphate calcineurin, which promotes DRP1 translocation to OMM and binding to the receptor (21). MFF/FIS1 are primary receptors located on the OMM and perform a vital role in recruiting DRP1 (22). Mitochondrial dynamics proteins of 49 kDa and 51 kDa (MID49 and MID51) also recruit and bind to DRP1, when MFF/FIS1 is not available (23). DRP1 is massively recruited and combined with the receptor, forming a ring-like structure to shear the mitochondria and promote the completion of mitochondrial fission (24). Mitochondrial fission usually occurs at the interface between mitochondria and the ER (**Figure 1**). This contact site is an extremely critical interface. It not only leads to the occurrence of mitochondrial fission but also is related to the rebuilds of mitochondrial cristae driven by transporting Ca^2+^ from the ER to the mitochondria (12).

The fission and fusion of mitochondria are dynamic and continuous processes. The balance between fission and fusion is very critical in maintaining the normal function of mitochondria. When the balance of mitochondrial dynamics is compromised, the mitochondrial dysfunction may disrupt normal metabolism through cytochrome C release, Ca^2+^ influx, excessive production of ROS, and mitochondrial protein efflux, causing cell damage and death (12, 25). For damaged mitochondria, however, mitochondrial fission can split this part of mitochondria out, and degrade or eliminate it through mitophagy pathways such as the PTEN-induced kinase 1 (PINK1)/Parkin or Bcl-2 19-kDa interacting protein 3 (BNIP3) (**Figure 1**) (26). In the process of resolving damaged mitochondria, mitochondrial fusion also exhibits a beneficial role by allowing the transmission of proteins, metabolites, and DNA across the network and attempting to restore and replenish mitochondrial function in exchange (27). Finally, mitochondrial fission, fusion, and mitophagy together operate the healthy mitochondrial pool. Disrupted mitochondrial dynamics would affect mitochondrial function and lead to the occurrence of diseases such as diabetic cardiomyopathy (28, 29).

## 3 Circadian clock and mitochondrial dynamics

### 3.1 Circadian clock

The circadian clock is temporal progress influenced by Earth’s rotation. Many activities of living organisms including gene expression (30), metabolism (31), immune and endocrine function (32, 33), as well as behavior (34), are controlled by day and night clocks. Circadian clock is composed of master pacemaker and peripheral clocks. The central, master clocks are located in the suprachiasmatic nucleus (SCN) of the hypothalamus. The peripheral clocks are virtually located in all the tissues and cells of the body (35). The circadian clock can synchronize internal 24-hour timing with a 24-hour solar day through a hierarchical network of master and peripheral oscillators.

**Figure 2:**
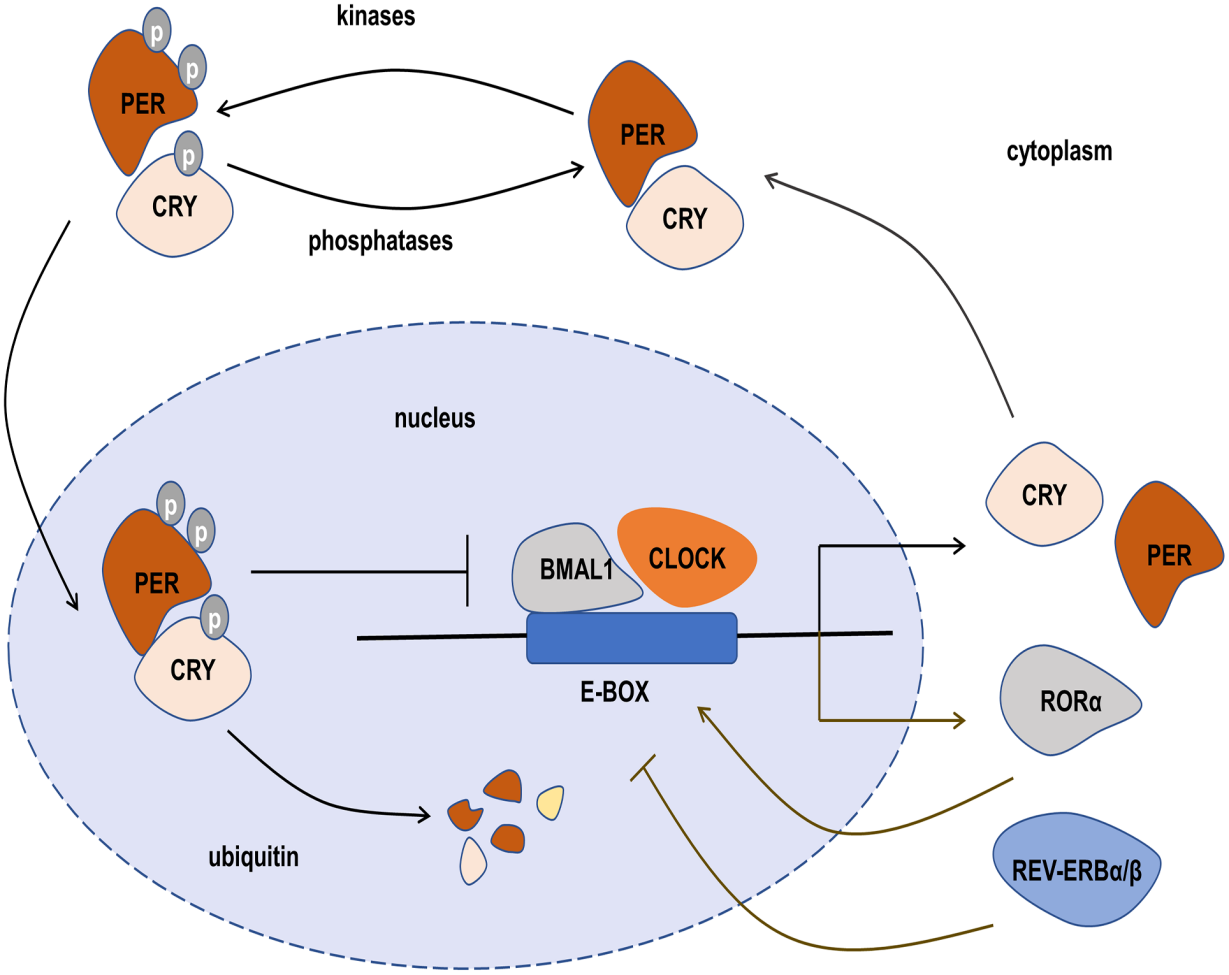
The transcription-translation feedback loop (TTFL) of the circadian clock. The main TTFL is driven by BMAL1-CLOCK dimer combined with E-box, and their negative regulators include the period (PER), cryptochrome (CRY), casein kinases, and phosphatases, which form a heterodimer in the cytoplasm and translocate into the nucleus to inhibit the transcription of CLOCK-BMAL1. In the second TTFL, REV-ERBα/β and RORα are also activated by CLOCK-BMAL1. REV-ERBα/β negatively regulates *BMAL1* transcription, but RORαpositively regulates *BMAL1* transcription.

The molecular circadian clock in mammals is formed by a transcription-translation feedback loop (TTFL). The main TTFL is driven by the transcription factors CLOCK-BMAL1 and their negative regulators including the period (PER) and cryptochrome (CRY), as well as some other regulators such as casein kinases (CKIα, CKIδ, and CKIϵ) and phosphatases (PP1, PP5), which regulate the stability and localization of these integral circadian proteins (36). The CLOCK-BMAL1 complexes directly combine with DNA to regulate E-BOX and induce the expression of negative regulators (37). The negative regulators PER and CRY form heterodimeric in the cytoplasm and translocate into the nucleus to inhibit the transcription of CLOCK-BMAL1. When the levels of PER and CRY decline through ubiquitin-dependent degradation, a new CLOCK-BMAL1-driven transcription cycle begins with 24-hour periodicity. The casein kinases and phosphatases also play a key role in the circadian period by controlling the activity of the PER-CRY dimer and the rate at which it enters the nucleus. In addition to the main feedback loop, the second feedback loop also plays an important role. The main components of the second feedback loop are the nuclear receptors REV-ERBα/β (38), retinoid-related orphan receptor α (RORα) (39), and CLOCK-BMAL1. Like the main loop, REV-ERBα/β and RORα are also activated by CLOCK-BMAL1. REV-ERBα/β negatively regulates *BMAL1* transcription, but RORα positively regulates *BMAL1* transcription (40). REV-ERBα/β and RORα compete for binding REV-​ERB–ROR response elements in the promoter and enhancer regions of the target gene, and make a rhythmic expression of the *BMAL1* gene (41). The primary function of the second feedback loop is to provide additional robustness to the oscillatory mechanism and counter surrounding disturbances to help circadian keep accurate timing (**Figure 2**) (36, 42).

The circadian clock is an internal and predictable mechanism that predicts the energy demands and metabolic changes through synchronization with light and temperature cycles (37). In this process, clock rhythm affects various cells or tissues, and confers various tissue-specific functions to circadian rhythmicity, such as the core body temperature with peak levels during the day and trough in the early morning, more oxidation delivered to the cell in active but less in inactive, and the melatonin secretion cycle that is inhibited by light (43). There is growing evidence that circadian disruption is involved in metabolic abnormalities. The liver-specific *BMAL1* knockout (KO) mice had higher levels of triglyceride, cholesterol, and free fatty acids than that in wild-type mice, and their livers contained lower levels of OXPHOS protein and complex I (10). Likewise, if the internal clock of an organism is uncoupled with the natural clock circadian, the individual will have deleterious effects on nutrient metabolism, such as increasing the risk of developing diabetes (44) and cardiovascular disease (45).

### 3.2 Effects of the clock on mitochondrial dynamics

The highly dynamic morphology of mitochondria is closely regulated by the circadian clock. It was shown that the mitochondria in cultured hepatocytes exhibited circadian alteration that the number of the tubular structure of mitochondria rhythmically decreased from the light to the dark (46). In cultured fibroblasts, the mitochondrial structure also showed rhythmic oscillations, a synchronous transformation from the tubular structure at 16 h after serum shock to a fragmented network at 28 h after serum shock (9). This rhythmic alteration of mitochondrial morphology is affected by circadian clock disruption. In the liver-specific *BMAL1* KO mice, the dynamic morphology of mitochondria lost rhythmic oscillation at different zeitgeber points (10). These mitochondria manifested bigger and rounder, and maintained a similar pattern throughout the day and night, whereas wild-type mice showed a cyclical change in morphology according to the surrounding changes (10).

**Figure 3:**
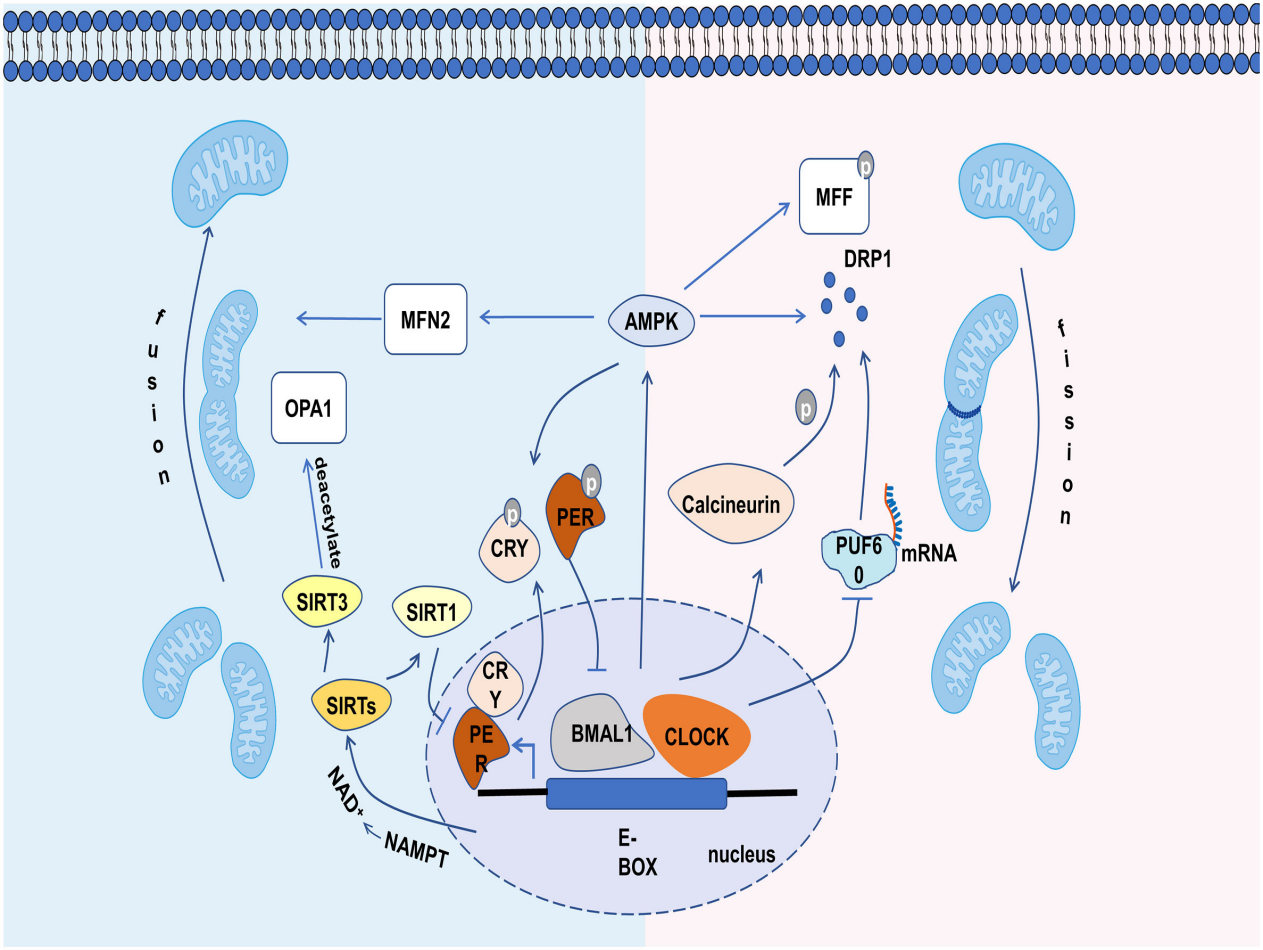
The relevant mechanisms by which circadian clock controls mitochondrial dynamics. The circadian clock regulates mitochondrial dynamics through influencing the molecules involved in it, including calcineurin, PUF60, AMPK, SIRT1 and SIRT3.

Aiming to illustrate the rhythmic changes in mitochondrial dynamics, the molecules related to mitochondrial dynamics have been studied in recent years. Calcineurin, which has a strong circadian rhythm (47), can dephosphorylate DRP1 at ser637 and promote the transfer of DRP1 from the cytoplasm to the mitochondrial membrane. Although the protein expression of calcineurin is constant throughout the day, its activation is under circadian regulation (47). Thus, the Ser637-phosphorylated DRP1 (P-DRP1) level exhibits circadian rhythm (9). The oscillations of DRP1 phosphorylation bring different levels of mitochondrial metabolism to adapt to environmental changes (9). It is also shown that CLOCK can accelerate the degradation of DRP1 mRNA through competitively inhibiting PUF60 function, a splicing factor that can improve DRP1 mRNA stability (48). The loss of CLOCK activity may release PUF60, resulting in increased DRP1 level and fragmented mitochondria (**Figure 3**) (48).

There are some other mediators linking mitochondria dynamics to the clock, such as AMP-activated protein kinase (AMPK), sirtuins (SIRTs) (9). AMPK is a serine/threonine kinase, whose activity, subunit composition, and localization depend on the circadian clock (49). For mitochondrial dynamics, AMPK not only enhances mitochondrial fission and mitophagy by phosphorylating MFF and recruiting DRP1 from cytoplasm under energy stress (50), but also increases mitochondrial fusion by increasing MFN2 expression during the fasting period, thereby improving the efficiency of ATP generation (51). In addition, the activated AMPK could phosphorylate PER2 and CRY, leading to the efficient expression of CLOCK and BMAL1 and shorting the period of the circadian clock (**Figure 3**) (52–54). SIRTs, a family of NAD^+^ (nicotinamide adenine dinucleotide)-dependent protein deacetylases, also play a significant role in the clock and mitochondrial dynamics. SIRT1 can promote the deacetylation and degradation of PER2 by binding to the CLOCK-BMAL1 heterodimers, thus participating in the regulation of circadian rhythm (55). Conversely, the CLOCK-BMAL1 can also influence the level of SIRT1 by regulating the gene expression of nicotinamide phosphoribosyl transferase (*NAMPT*), an important enzyme for the production of NAD^+^ (56). Recent studies have shown that the circadian clock can regulate the level of deacetylated OPA1 through the NAD^+^/SIRT3 pathway (**Figure 3**) (54). Cardiac OPA1 is hyperacetylated during pathological stress, and this modification reduces its activity of GTPase, resulting in mitochondrial fusion disorder (57). SIRT3 can deacetylate and activate OPA1, and eventually restore mitochondrial dynamics (57). Overall, mitochondrial dynamics are closely controlled by the clock and exhibit a circadian rhythm.

## 4 Clock-controlled mitochondrial dynamics in DCM

**Figure 4:**
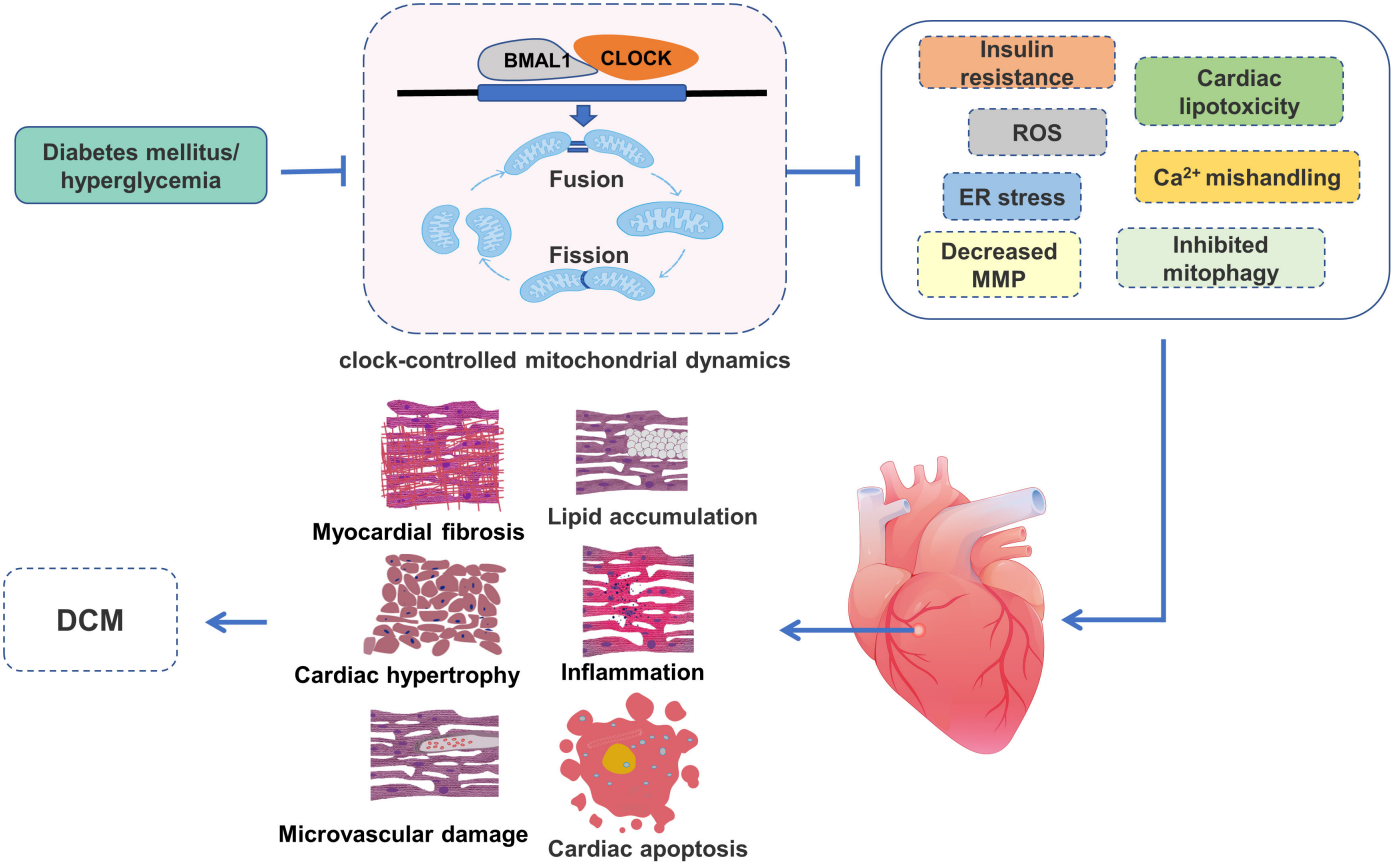
The role of clock-controlled mitochondrial dynamics in DCM. The disturbed clock-controlled mitochondrial dynamics may affect insulin signaling, lipid metabolism, mitochondrial ROS production, Ca^2+^ processing, MMP, mitophagy and ER stress, resulting in lipid accumulation, inflammation, myocardial fibrosis, cardiac hypertrophy, cardiac apoptosis and microvascular damage, and ultimately participate in the development of DCM.

The clock-controlled mitochondrial dynamics are susceptible to energy stress. Many studies have reported that circadian clock and mitochondrial dynamics are disordered in the diabetic state (58–61). The disordered clock-controlled mitochondrial dynamics adversely affect cardiomyocytes through several underlying mechanisms, including insulin resistance, cardiac lipotoxicity, ROS, mitochondrial Ca^2+^ mishandling, decreased MMP, impaired mitophagy and ER stress, which ultimately lead to the development and progression of DCM (**Figure 4**).

### 4.1 Clock-controlled mitochondrial dynamics and insulin resistance

Insulin resistance is a critical pathophysiological abnormality associated with DCM. Insulin resistance has many detrimental effects on cardiomyocytes, such as decreased glucose uptake, elevated lipid metabolites, and increased glycation reactions. These harmful effects may lead to myocardial stiffness, reduced ejection fraction, and heart failure (62). Insulin resistance is closely related to mitochondrial dynamics disorder. In insulin-resistant cardiomyocytes, mitochondrial dynamics are disordered, manifested as decreased MNF1-mediated mitochondrial fusion and increased DRP1-mediated mitochondrial division, resulting in mitochondrial dysfunction and excessive ROS production (63). The disordered mitochondrial dynamics also affect insulin secretion and insulin signaling pathways. The overexpression of MFN1 or MFN2 can promote mitochondrial fusion, accompanied by improvement of insulin receptor substrate 1 (ISR1)-Akt signaling and insulin-stimulated glucose absorption (64). On the contrary, promoting mitochondrial division by overexpression of DRP1 and FIS1 show the opposite behavior (64). MFN1/2 are also demonstrated to be pivotal for glucose-stimulated insulin secretion by controlling mitochondrial DNA content and may be promising targets to restore glucose control in diabetes (65). Thus, mitochondrial dynamics play a critical role in the physiological function of insulin by impacting the insulin signaling pathway and insulin secretion.

It is worth noting that insulin resistance is closely related to circadian alteration. Under normal physiological conditions, insulin secretion and insulin receptor sensitivity have a certain rhythm, that is insulin secretion increasing during the day and decreasing during the night, and insulin receptor sensitivity reaching a peak during the day (66, 67). Nonetheless, the mice with circadian rhythm disturbances induced by BMAL1 and CLOCK mutations showed impaired glucose tolerance, reduced insulin secretion, and damaged islet size and proliferation, which progressively worsened with age (44). The insulin signaling pathways are also influenced by the circadian system. A large number of insulin signaling proteins (e.g. IRS1, Pik3r1, Akt1, and Akt2) in cardiomyocytes from clock mutant mice are significantly reduced compared with that in wild-type mice, which is consistent with the decreased insulin regulation of glucose metabolism (68). Recently, by using chromatin immunoprecipitation sequencing, researchers have found that CLOCK and BMAL1 could bind to inner-mitochondrial genes related to insulin sensitivity, suggesting that the relationship between circadian rhythm and insulin resistance may be related to mitochondrial dynamics (69). Jacobi and his colleagues also observed insulin resistance when they studied changes in mitochondrial dynamics by knocking out the *BMAL1* gene (10). Consistently, Ye et al. observed that BMAL1 inhibition resulted in mitochondrial dynamics disorder, as well as impaired insulin signaling in pancreatic beta cells (70). Thus, mitochondrial dynamics are inherently influenced by clock rhythm, and the disordered clock-controlled mitochondrial dynamics may lead to insulin resistance and promote DCM.

### 4.2 Clock-controlled mitochondrial dynamics and cardiac lipotoxicity

Lipid accumulation is a common feature of the diabetic heart. ​Lipids that exceed the storage and oxidation capacity of the heart may produce a variety of lipotoxic intermediates, including ceramides, diacylglycerol, and oxidized phospholipids, which are detrimental to cardiac morphology and function (71). The lipotoxic intermediates contribute to the development of DCM by triggering cellular signaling (such as cellular metabolism, growth, and proliferation) and modifications of proteins and lipids (72). It is worth noting that the accumulation of lipids and the production of lipotoxic intermediates in the heart is associated with abnormal mitochondrial dynamics (73). Carnitine palmitoyl transferase 1 (CPT1) is the rate-limiting enzyme of β-oxidation that determines the rate of fatty acid entry into mitochondria. Wang et al. have shown that the abnormal activation of DRP1 in mice deficient in low-density lipoprotein receptor-related protein 6 could inhibit the activity of the CPT1 transcription factors CTCE and c-Myc, leading to fatty acid accumulation and heart failure (74). Ceramides are more directly related to mitochondrial dynamics. A large accumulation of ceramides may lead to mitochondrial fragmentation and mitochondrial apoptotic pathways, thereby promoting cell death and insulin resistance (75, 76). Recent studies have shown that the increased ceramide synthase 6, a key enzyme in ceramide synthesis, is associated with fragmented mitochondria and insulin resistance in high-fat diet induced obese mouse models (77, 78), and that inhibition of ceramide synthase 6 improves mitochondrial function and insulin signaling (78). Similarly, downregulation of DRP1 decreases mitochondrial fission and protects H9C2 cells from lipotoxicity (79). These findings provide evidence that abnormal mitochondrial dynamics are closely associated with cardiac lipotoxicity in diabetes.

It is not surprising that the lipid uptake and oxidation are controlled by circadian clock. Under physiological conditions, the myocardium rhythmically takes up and utilizes lipid in response to variable environmental conditions (80). Disruption of circadian rhythms induced by genetic or environmental perturbation results in abnormal cardiac lipid metabolism, imbalances in lipid availability, and lipid oxidation rates, leading to the accumulation of intercellular lipotoxic derivatives (81, 82). Peroxisome proliferator-activated receptor (PPAR) α is a master nuclear receptor, which plays a critical role in lipid metabolism through regulating lipid transport, esterification, and oxidation. Abnormal expression of PPARα in the heart is thought to be an important player in cardiac lipotoxicity (83). This conception is supported by previously published experimental data showing that overexpression of PPARα leads to lipid accumulation and the development of DCM (84), and that pharmacological inhibition of PPARα reduces cardiac lipotoxicity in diabetes (84, 85). Recent studies have shown that the *PPARα* gene can be transactivated by the CLOCK/BMAL1 heterodimer *via* an E-BOX-dependent mechanism (86). The mutation of BMAL1/CLOCK increases PPARα mRNA N6-methyladenosine, which affects PPARα stability and increases lipid accumulation (86–88), suggesting that the circadian clock is also involved in lipid metabolism. However, under diabetic condition, the disturbed circadian clock is frequently accompanied by disordered mitochondrial dynamics and cardiac lipotoxicity (71, 89, 90). Collectively, currently available research findings suggest that restoring clock-controlled mitochondrial dynamics may be an effective way to reduce DCM by reducing cardiac lipotoxicity.

### 4.3 Clock-controlled mitochondrial dynamics and mitochondria-generated ROS

The role of mitochondria-generated ROS in DCM is well-established (91). It is well demonstrated in diabetes that cardiomyocytes from animal models and patients display mitochondrial dysfunction and overproduction of ROS (92–94). Large amounts of ROS result in the damage of cardiomyocyte proteins, lipids, and DNA, eventually leading to the development of DCM (91). It is worth noting that abnormal mitochondrial morphology is intricately linked to the excessive production of ROS. Increasing studies have shown large amounts of fragmented mitochondria in diabetic cardiomyocytes, accompanied by excessive ROS production, which is reduced by inhibition of mitochondrial fission through decreasing DRP1 expression (95, 96). Additionally, improving OPA1 expression could decrease mitochondrial ROS generation by stabilizing oligomers and activity of ATPase, which is a key enzyme in ATP generation using electron potential energy to produce ATP (28, 97). Notably, the excessive mitochondrial ROS in the heart could promote mitochondrial fission by decreasing DRP1 phosphorylation and decrease mitochondrial fusion by altering OPA1 hydrolysis (98). Thus, it is understandable that the fragmented mitochondria induced by hyperglycemia can be restored with ROS scavenger (99). Therefore, the disordered mitochondrial dynamics and excessive ROS production may interact with each other, and it is a vicious cycle in diabetes.

ROS production in the process of mitochondrial metabolism in the physiological state has a circadian oscillation rhythm. Mitochondria generate more ROS at sleep onset relative to the wake period, due to higher levels of the clock genes BMAL1 and CLOCK at sleep onset (100). A recent study has indicated the decay of circadian genes (PER, TIM, CLOCK) oscillation with elevated ROS levels in diabetes (101). The disruption of clock components can impact ROS production. In the pancreatic β-cell line, BMAL1 knockdown triggers an increase of the ROS content and impairs glucose-stimulated insulin secretion, the hallmark of the pancreas islet function in diabetes (102). Similarly, the loss of *CLOCK* gene increased ROS production and impaired cardiac structure and function (103). PER1-deficient mice also show impaired ROS-production rhythm with lower glutathione peroxidase activity and higher ROS level (104). In addition to affecting ROS production, the circadian clock also affects the anti-oxidative system. BMAL1 interacts with HSPB1, a small heat shock protein that resists ROS *via* S-thiolated modification, to reduce oxidative damage in cardiomyocytes (105). PER interacts with glutathione peroxidase to withstand oxidative stress, and reduced PER expression will diminish the activity of glutathione peroxidase (104). Recent studies have indicated that a disordered circadian clock may increase ROS generation or weaken antioxidant activity and is often accompanied by mitochondrial dynamics dysfunction. The disruption of functional *CLOCK* gene in cardiac myocytes impaired the expression of mitochondrial fusion proteins OPA1 and MFN2, resulting in fragmented mitochondria and the accumulation of mitochondrial-generated ROS (103). Further study indicated the CLOCK could improve mitochondrial dynamics and function by stabilizing DRP1 mRNA expression (48). In CLOCK mutation mice, abnormal accumulation of DRP1 leads to fragmented mitochondria and increased ROS levels (48). Together, these results suggest that mitochondrial-generated ROS is impacted by circadian-controlled mitochondrial dynamics and plays an important role in DCM.

### 4.4 Clock-controlled mitochondrial dynamics and mitochondrial Ca^2+^ handling

The abnormal Ca^2+^ signaling is a critical feature of DCM, notably mitochondrial Ca^2+^ mishandling (106). Mitochondrial Ca^2+^ handling normally provides a basis for normal excitation-contraction coupling and mitochondrial energy supply, but the disordered Ca^2+^ concentration in the mitochondrial matrix in diabetes contributes to decreased ATP generation and increased cardiomyocyte damage (8, 106, 107). Mitochondrial Ca^2+^ mishandling is affected by abnormal mitochondrial dynamics. In cultured cardiomyocytes, FIS1-induced mitochondrial fragmentation could reduce mitochondrial Ca^2+^ uptake (108). A similar observation is made later in the myofibers, where the inhibition of DRP1 could increase Ca^2+^ concentration in the mitochondrial matrix during the phase of electrical stimulation, and increase the expression of mitochondrial calcium uniporter (MCU), an important channel for mitochondrial Ca^2+^ uptake (109). The mitochondria-associated ER membrane (MAM), a critical regulator affecting mitochondrial Ca^2+^ handling, is also demonstrated to be affected by mitochondrial dynamics. In fly fruit hearts, MFN2 deficiency impair the physical contact between mitochondria and ER and decrease the protein content associated with MAM, resulting in reduced mitochondrial Ca^2+^ uptake (110). A further study has also shown that the disordered MAM induced by MFN2 knockdown impairs ER Ca^2+^ release and decreases ATP generation in ventricular myocytes (111). Additionally, the mutation of OPA1 could reduce the distance between ER and mitochondria, suggesting OPA1 is necessary for mitochondrial Ca^2+^ handling (112).

Notably, mitochondrial Ca^2+^ handling itself has circadian oscillations and is controlled by clock system. A recent study applied calcium pulses to isolate mitochondria in mouse hearts at different periods to assess the calcium retention capacity and calcium uptake rate of mitochondria, showing mitochondria have higher calcium retention capacity and calcium absorption rate during the sleep stage (100). For some critical proteins of mitochondrial Ca^2+^ handling, MCU and sodium/calcium exchanger had higher expression levels at sleep onset relative to the wake phase (100). And, other proteins participated in mitochondrial Ca^2+^ handling are also influenced by the circadian clock, such as ryanodine receptors (RyR). The circadian complex CLOCK/BMAL1 can bind to the E-box of the RyR gene and impact its gene expression (113). In the suprachiasmatic nucleus of BMAL1 KO mice, the levels of *RyR* mRNA and RyR protein are significantly reduced, as well as the decreased intracellular Ca^2+^ concentration (114). In the models of disordered circadian clock, dysfunctional mitochondrial dynamics and mitochondrial Ca^2+^ mishandling are well observed (9, 100). Hyperglycemia is inherently a critical player, causing circadian clock disturbance, dysfunctional mitochondrial dynamics and mitochondrial Ca^2+^ mishandling (89, 90, 106). These findings provide important insights that clock-controlled mitochondrial dynamics may regulate mitochondrial Ca^2+^ handling and influence the development of DCM.

### 4.5 Clock-controlled mitochondrial dynamics and mitochondrial membrane potential

Mitochondria produce ATP through the electron transport chain, which creates an electrochemical gradient and generates mitochondrial membrane potential (MMP) (115). MMP is an important parameter for evaluating mitochondrial function and activity (116). Normal MMP is the key condition for mitochondrial oxidative phosphorylation, and its stability contributes to maintaining normal physiological function of the cells (115, 117). The abnormality of MMP may be attributable to apoptosis, ROS, and abnormal autophagy (118, 119). Mitochondrial dynamics is closely linked to MMP. In diabetic hearts, both abnormal MMP and dysfunctional mitochondrial dynamics are commonly observed. Diabetic hearts from db/db mice show excessive fragmented mitochondria and decreased MMP. Increased fusion events through reconstituting MFN2 in DCM restored the MMP and mitochondrial function (120). The lipotoxic hearts also exhibit impaired mitochondrial fusion due to decreased OPA1 expression and abnormal MMP (98). Further studies have revealed that the specific mechanism by which OPA1 changes MMP may be related to changes in mitochondrial cristae architecture (117). Moreover, the loss of MMP destabilizes the L-OPA1 structure, leading to increased OPA1 cleavage and consequent impact on mitochondrial fusion (121). Likewise, in cardiomyocytes treated with high glucose, the inhibitors of DRP1, Mdivi-1, not only decreased excessive mitochondrial fission but also alleviated the decreased MMP (122).

MMP is also influenced by the circadian clock. Under normal physiological conditions, MMP has certain oscillatory patterns and is also associated with mitochondrial activity. A study shows that, in the suprachiasmatic nucleus of rats, MMP is higher during the light period than the dark period (123). In semi-anaerobic yeast cells, MMP is oscillating, and this oscillation may be related to mitochondrial metabolic activity (124). Further research indicates that this oscillatory pattern correlates with mitochondrial dynamics, mitochondrial fusion along with high MMP (125). Disruption of circadian rhythm leads to abnormal MMP and imbalance in mitochondrial dynamics. In pancreatic beta cells, the loss of BMAL1 decreased the expression of MNF1/2 and increased the expression of FIS1, accompanied by decreased MMP and impaired pancreatic function (70). Similarly, CLOCK-deficient cardiac myocytes showed excessive mitochondrial fission, loss of MMP, and impaired cardiac structure (103). Therefore, it can be speculated that the disordered clock rhythm under diabetic conditions can lead to the dysfunction of mitochondrial dynamics and the decrease of MMP, thereby increasing myocardial damage.

### 4.6 Clock-controlled mitochondrial dynamics and mitophagy

Hyperglycemia can easily lead to mitochondrial damage in diabetes. The progressive mitochondrial damage in cardiomyocytes leads to lipid accumulation and excessive oxidative stress, resulting in the development of DCM (126). To prevent damage to dysfunctional mitochondria, a selective degradation system referred to as “mitophagy” is activated to remove dysfunctional mitochondria (126). However, increasing studies demonstrate impaired mitophagy in diabetes (127, 128). Although the molecular mechanism of impaired mitophagy in DCM has not been fully clarified, recent findings imply that abnormal mitochondrial dynamics are involved. PINK/Parkin are critical molecules in ubiquitin-dependent mitophagy (129). In mouse cardiomyocytes, the OMM guanosine triphosphatase MFN2 could mediate Parkin recruitment to the damaged mitochondria *via* a PINK-dependent manner (130). Downregulation of MNF2 expression prevents depolarization-induced translocation of Parkin to the damaged mitochondria and inhibits mitophagy (130), which contributes to the development of DCM (120). Likewise, DRP1 disruption in cardiomyocytes leads to imbalanced mitochondrial dynamics, and suppresses mitophagy *via* reducing the formation of autophagosomes, resulting in cardiac dysfunction (131). In cultured H9C2 cardiomyocytes, the overexpression of OPA1 promotes mitochondrial fusion and stimulates mitophagy, thereby attenuating high glucose-induced cardiomyocytes injury (132). These findings suggest that mitochondrial dynamics are involved in the regulation of mitophagy and thus influence the development of DCM.

Mitophagy is also demonstrated to be regulated by circadian clock. The key regulators of mitophagy, PINK/Parkin, are rhythmically expressed under clock control in response to environmental changes (103). The disruption of clock rhythm reduces the expression levels of PINK/Parkin, leading to suppressed mitophagy and myocardial dysfunction (103). BNIP3 is one of the important molecules of receptor-dependent mitophagy (129). It has been shown that BMAL1 binds to the E-BOX element in the promoter region of BNIP3 gene and regulates the level of BNIP3 protein oscillation in human embryonic stem cell-deprived cardiomyocytes (133). Downregulating BMAL1 expression directly reduces BNIP3 expression, leading to compromised mitophagy and cardiomyocyte dysfunction (133). Recently, Jacobi et al. have indicated that mitochondrial fission and mitophagy proteins show a similar diurnal pattern in the livers of WT mice (10). However, their levels fail to cycle and are significantly reduced in BMAL1 KO mice, and mitochondrial fission and mitophagy-related genes *DRP1, FIS1, PINK1*, and *BNIP3* are also greatly reduced, manifested by fragmented mitochondria and suppressed mitophagy (10). In cardiomyocytes, similarly, the loss of CLOCK/BAML1 expression may impair mitochondrial dynamics and suppress mitophagy, leading to dysfunctional mitochondria and cardiac injury (103, 133). Thus, the disordered circadian clock-controlled mitochondrial dynamics contributes to compromised mitophagy, which promotes the development of DCM.

### 4.7 Clock-controlled mitochondrial dynamics and ER stress

The main function of ER is protein folding and assembly. Hyperglycemia and hyperlipidemia in diabetes impair ER function, leading to the accumulation of unfolded proteins (134). This process can be prevented by a quality control system termed unfolded protein response (UPR), a signal transduction pathway alleviating the accumulation of abnormal proteins in the ER lumen (135). If the UPR is not able to process these unfolded proteins within a certain time lapse, it will induce cardiomyocyte apoptosis and promote the progress of DCM (136). In animal models of diabetes, ER stress has been shown to contribute to cardiac apoptosis, as evidenced by the induction of UPR signaling proteins and ER stress-associated apoptosis signaling proteins (71). UPR mainly contains three signaling pathways, protein kinase RNA-like ER kinase/activating transcriptional factor 4 (PERK/ATF4), inositol-requiring protein1α/X-box binding protein (IRE1α/XBP1), and activating transcriptional factor 6 (ATF6) pathways (134), which are demonstrated to be influenced by mitochondrial dynamics. The overactivated UPR branches such as PERK and ATF6 in hyperglycemia-treated cardiomyocytes can be alleviated by reducing MFN2 expression (137), and downregulation of MFN2 could attenuate mitochondrial dysfunction and ER-stress induced cardiomyocytes apoptosis (138). An opposite finding performed by other researchers suggests that MNF2 can suppress PERK activity through direct interaction under basal conditions, but hyperglycemia inhibits MFN2 expression and promotes the reduction of MNF2-PERK interaction, thereby MNF2 overexpression could alleviate the abnormal activation of PERK pathway, cardiomyocytes apoptosis and mitochondrial dysfunction (139, 140). In addition, DRP1 is also involved in ER stress- induced pancreatic β-cell apoptosis through the process of mitochondrial fission, cytochrome c release, ROS generation and caspase-3 activation (141).

It is worth noting that many proteins related to UPR and ER stress are closely regulated by circadian rhythms (142). The circadian clock can coordinate the rhythmic activation of IRE1α in 12-hour cycles to respond to the metabolic demands of organism. However, in Cry1/Cry2 KO mice liver, the rhythmic activation of IRE1α was lost, accompanied by disrupted lipid metabolism (143). A similar study was conducted in *CLOCK* mutant mice, showing the UPR-related genes in liver such as *PERK, IRE1α, and ATF6*, were significantly up-regulated as compared to the WT mice (142). Additionally, the disruption of circadian rhythms caused by sleep deprivation or gene mutant in pancreas contributed to ER stress, resulting in the loss of pancreatic beta-cells and the development of diabetes (144, 145). Recent studies have shown that disturbing circadian clock can lead to ER stress and affect UPR-related protein expression, accompanied by disturbed mitochondrial dynamics (145, 146). Considering that the circadian clock is inherently susceptible to hyperglycemia (89), and that there exists disturbed mitochondrial dynamics and ER stress in diabetic myocardium (90, 134), it can be speculated that restoring the clock-controlled mitochondrial dynamics may inhibit ER stress and alleviate the development of DCM.

## 5 Clock-controlled mitochondrial dynamics as a novel therapeutic target in DCM

There is a lack of effective therapeutic approaches for DCM due to its complex etiology. However, glycemic control is still the core part. In healthy subjects, the combination of basal glucose production and insulin-mediated suppression of glucose production, in alliance with oscillatory insulin levels, keeps glycemia stable throughout the day (147). The glycemia in patients with diabetes has a special oscillatory pattern, manifested as increased glycemic variability with specific circadian characteristics (148, 149). Epidemiological evidence suggests that irregular eating habits, reduced light exposure, increased night shift hours, nocturnal light exposure, and sleep deprivation disrupt the circadian clock and increase the risk of diabetes (150, 151). Thus, it is understandable that enhancing circadian clock rhythms through adjusting lifestyle contributes to prevention and treatment of diabetes-associated complications (152).

The potential novel drugs targeting clock-controlled mitochondrial dynamics are also imminent. The first circadian clock-based drug was melatonin, a hormone secreted by the pineal gland (153). Exogenous melatonin supplementation increased ROR levels in diabetic myocardium, and prevented the development of DCM *via* reducing dysfunctional mitochondria, ER stress, myocardial apoptosis, autophagy dysfunction, and oxidative stress damage (60, 153, 154). In recent years, a number of small-molecule chemical enhancers targeting the circadian system have been developed, such as Nobiletin, CRY activator (KL001), Rev-ERB-α/β agonist (especially SR9011/SR9009), and so on (​The mechanism of their action is given in **Table 1**). These chemical compounds restored cardiac circadian rhythms and oscillatory patterns of metabolic gene expression, resulting in phenotypical improvements in insulin resistance, lipotoxicity, oxidative stress, and dysfunctional mitochondrial dynamics (153, 155–158, 161–163). Notably, improving mitochondrial dynamics is also a critical approach to restore myocardial function in diabetes. It is well demonstrated that inhibiting mitochondrial fission or increasing mitochondrial fusion has beneficial effects on diabetic hearts. Ding et al. suggest that the administration of mitochondrial fusion promoter-M1 can restore mitochondrial dynamics balance and attenuate DCM *via* an OPA1-dependent way (28). Similarly, Hu et al. have shown that reconstitution of MFN2 in diabetic myocardium inhibits mitochondrial fission and prevents DCM progress (120). In a mouse model of diabetes, increasing MFN2 expression could improve mitochondrial function, inhibit mitochondrial oxidative stress, and reduce cardiomyocyte apoptosis (160). In addition, melatonin attenuates the development of diabetes-induced cardiac dysfunction by preventing DRP1-mediated mitochondrial fission through the SIRT1-PGC1α pathway (164). Therefore, the interventions involving in circadian clock and mitochondrial dynamics are promising approaches for the treatment of DCM (The summary of potential drugs were shown in **Table 1**).

**Table 1:** Summary of potential interventions involving clock-controlled mitochondrial dynamics in DCM.

Potential drugs	Effects on clock circadian/mitochondrial dynamics	Other effects on DCM	References
Melatonin	Increase RORα expression Increase DRP1 expression	Decrease myocardial apoptosis Reduce oxidative stress Mitigate myocardial hypertrophy and cardiac fibrosis Improve cardiac diastolic function	(60, 153, 155)
Nobiletin	Enhance amplitude of circadian rhythms Enhance RORα/γ transcriptional activity Increase BMAL1 expression	Improve glucose tolerance and insulin sensitivity Increase insulin secretion	(155, 156)
KL001	Increase CRY level	Suppress glucose production	(157)
SR9011/ SR9009	Increase REV-ERBα/β expression	Decrease triglyceride synthesis Increase lipid and glucose oxidation Mitigate cardiac fibroblasts	(158, 159)
Mitochondrial fusion promoter-M1	Increase OPA1 expression	Improve mitochondrial function Inhibit myocardial apoptosis Decrease ROS generation	(28)
Nicotinamide riboside	Increase MFN2 expression	Reduce cardiomyocyte apoptosis Decrease ROS generation	(120, 160)

## 6 Conclusion

Circadian clock-controlled mitochondrial dynamics are critical for the normal structure and function of the heart. Alternations in the circadian clock and mitochondrial dynamics in diabetes play an important role in the pathophysiological process of DCM. In this review, we summarize the relevant pathways of circadian clock-controlled mitochondrial dynamics and discuss how the disruption of circadian clock and mitochondrial dynamics impact multiple etiologies of DCM, including insulin resistance, cardiac lipotoxicity, mitochondria-generated ROS, mitochondrial Ca^2+^ handling, MMP, mitophagy, and ER stress. This study also provides a strong rationale for targeting the circadian clock and mitochondrial dynamics in the treatment and prevention of DCM. Further studies are urgently needed to identify and characterize the mechanisms of action of novel chemical and endogenous modulators of the circadian clock and mitochondrial dynamics to prevent heart damage in diabetic states.

## Author contributions

ZJ, YJ, and WS wrote the manuscript and performed the validation. LZ, XJW, LG, and JG carried literature research. YL, YZ, XYW, and Z-YX performed manuscript review. ZX and SL contributed to the conception of the manuscript and contributed significantly to manuscript preparation. All authors contributed to the article and approved the submitted version.
